# Analysis of *Fibroblast Growth Factor 14* (*FGF14*) structural variants reveals the genetic basis of the early onset nystagmus locus NYS4 and variable ataxia

**DOI:** 10.1038/s41431-022-01197-5

**Published:** 2022-10-07

**Authors:** Fabiola Ceroni, Daniel Osborne, Samuel Clokie, Dorine A. Bax, Emma J. Cassidy, Matt J. Dunn, Christopher M. Harris, Jay E. Self, Nicola K. Ragge

**Affiliations:** 1 Faculty of Health and Life Sciences, Oxford Brookes University, Oxford, UK; 2 Department of Pharmacy and Biotechnology, University of Bologna, Bologna, Italy; 3 Clinical and Experimental Sciences, Faculty of Medicine, University of Southampton, Southampton, UK; 4 West Midlands Regional Clinical Genetics Service and Birmingham Health Partners, Birmingham Women’s and Children’s Foundation Trust, Birmingham, UK; 5 Wessex Regional Genetics Laboratory, Salisbury NHS Foundation Trust, Salisbury District Hospital, Salisbury, UK; 6 School of Optometry and Vision Sciences, Cardiff University, Cardiff, UK; 7 Royal Eye Infirmary, Derriford Hospital, Plymouth, UK

**Keywords:** Disease genetics, Movement disorders

## Abstract

Nystagmus (involuntary, rhythmical eye movements) can arise due to sensory eye defects, in association with neurological disorders or as an isolated condition. We identified a family with early onset nystagmus and additional neurological features carrying a partial duplication of *FGF14*, a gene associated with spinocerebellar ataxia type 27 (SCA27) and episodic ataxia. Detailed eye movement analysis revealed oculomotor anomalies strikingly similar to those reported in a previously described four-generation family with early onset nystagmus and linkage to a region on chromosome 13q31.3-q33.1 (NYS4). Since *FGF14* lies within NYS4, we revisited the original pedigree using whole genome sequencing, identifying a 161 kb heterozygous deletion disrupting *FGF14* and *ITGBL1* in the affected individuals, suggesting an *FGF14*-related condition. Therefore, our study reveals the genetic variant underlying NYS4, expands the spectrum of pathogenic *FGF14* variants, and highlights the importance of screening *FGF14* in apparently isolated early onset nystagmus.

## Introduction

Congenital and early onset nystagmus (involuntary, repetitive oscillation of the eyes) typically manifests within the first months of life. It can be apparently isolated, associated with visual deficits, or seen in the context of numerous neurological disorders. Given the genetic and clinical heterogeneity of these conditions, detailed visual and neurological phenotyping, with analysis of supranuclear eye movements, can direct clinicians towards the underlying genetic causes [1, 2]. However, typical patterns of clinical features suggesting an underlying cause, such as those observed in Infantile Nystagmus Syndrome (INS) or cerebellar-type nystagmus, are not always present [3]. Whole-scale genetic testing is now assisting in diagnosing complex disorders such as nystagmus and, as described here, redefining phenotypes associated with individual gene-related conditions.

Here, we describe a father and son with nystagmus, early onset tremor, and motor difficulties, including mild ataxia. Array-CGH revealed that both individuals carry a partial duplication of *FGF14* (*Fibroblast Growth Factor 14*, OMIM: 601515). Heterozygous *FGF14* variants are associated with spinocerebellar ataxia type 27 (SCA27) [4] and episodic ataxia (EA) [5], although some individuals display milder phenotypes, including tremor without ataxia [5] or nystagmus with occasional episodes of vertigo and incoordination [6]. Detailed eye movement analysis revealed oculomotor anomalies strikingly similar to those described in a large dominant pedigree with linkage to a locus on chromosome 13q31.3-q33.1 (NYS4, OMIM: 193003) [7, 8], containing *FGF14*. Herein, we revisited the original NYS4 pedigree and identified a heterozygous deletion disrupting *FGF14* and *ITGBL1* (*Integrin Subunit Beta Like 1*, OMIM: 604234), segregating with the disorder. Therefore, this study determines the genetic variant underlying NYS4 and highlights the importance of *FGF14* structural variants in milder forms of SCA27, including apparently isolated childhood nystagmus.

## Cases and methods

Families 1 and 2 were recruited to a national ‘Genetics of Eye and Brain Anomalies study’ (REC 04/Q0104/129). Informed consent was obtained according to the tenets of the Declaration of Helsinki.

Family 1: Copy Number Variant (CNV) screening was performed using a 60-mer oligo-array (8x60K International Standard Cytogenomic Array [ISCA] Consortium configuration [Oxford Gene Technology, Oxford, UK]). Paternal DNA was sequenced with an Illumina HiSeq and SureSelect Ataxia Panel v1 including *FGF14* (Agilent Technologies, Santa Clara, CA, USA).

Family 2: Whole genome sequencing (WGS) was performed using paired-end, 2 × 150, and 30x coverage with an Illumina NovaSeq 6000 (Theragen Bio, Republic of Korea). The presence of sequence variants in diagnostic ataxia or nystagmus genes was assessed (PanelApp panels “Hereditary ataxia and cerebellar anomalies - childhood onset” v6.28, “Albinism or congenital nystagmus” v1.5, “Infantile nystagmus” v1.3; https://panelapp.genomicsengland.co.uk/). Structural variants were identified using bbmap (https://sourceforge.net/projects/bbmap/). Breakpoints were identified from bbmap-aligned files using the GRIDSS package [9] and validated by PCR and Sanger sequencing.

Both CNVs were evaluated according to the ACMG guidelines [10] using the ClinGen CNV Interpretation Calculator (https://cnvcalc.clinicalgenome.org/cnvcalc/).

## Results

### Family 1

**Fig. 1:**
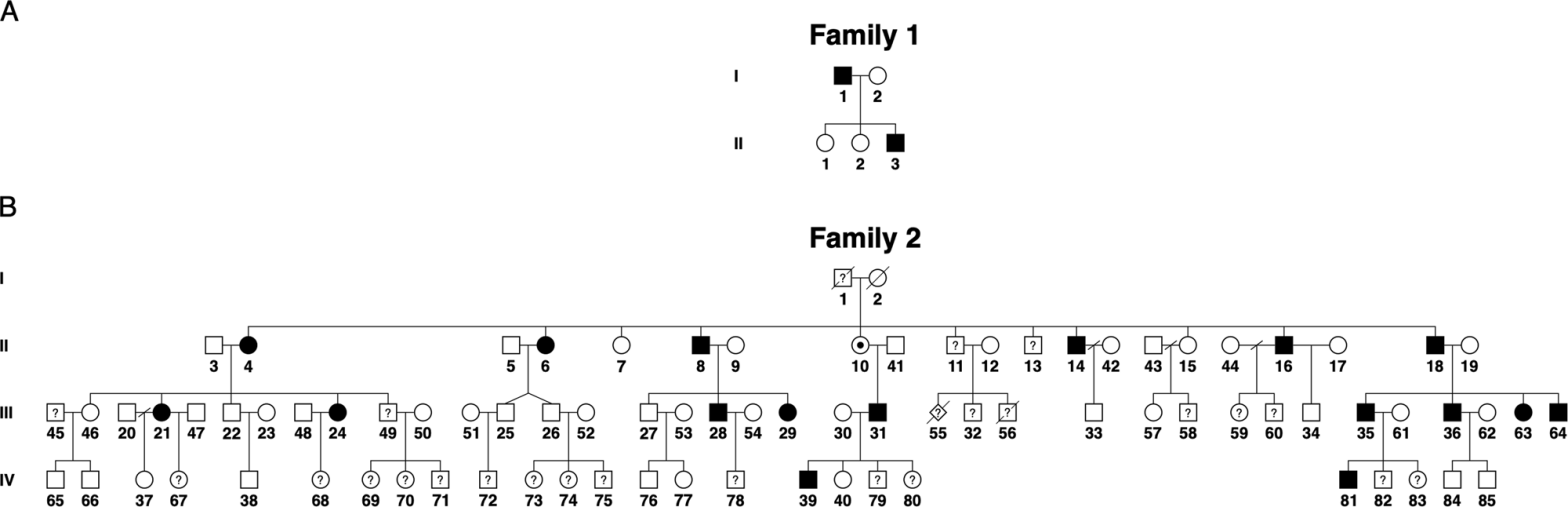
Pedigrees of the two families with *FGF14* structural variants. **A** Pedigree of family 1. The proband (II.3) is the third child of nonconsanguineous parents. Black-filled symbols indicate a SCA27 phenotype. **B** Pedigree of family 2, with individuals numbered according to recruitment order. Black-filled symbols represent individuals with eye movement anomalies. Individuals III.63 and III.64 were assessed through a video call. Question marks indicate individuals with affected status unknown. The obligate carrier status of individual II.10 is indicated by a black dot.

An 8-year-old boy (II.3, Fig. 1A) was referred to the eye clinic with apparently isolated nystagmus since age 4 years. History and clinical examination revealed that he had mild developmental delay and had started walking after age 2 years. His visual acuity was within normal range (logMAR < 0.18 either eye). He had vertical upbeat nystagmus in primary position, horizontal gaze-evoked nystagmus in side gazes and horizontal rebound nystagmus. Eye movement recordings showed that horizontal and upward smooth pursuits were absent, but downward smooth pursuits were present with reduced gain. His electroretinogram (ERG), visual evoked potentials (VEPs), and cranial magnetic resonance imaging (MRI) were normal. Subsequent neurological examination identified bilateral intention tremor, mild dysmetria, dysdiadochokinesis, and difficulties with heel-to-toe walking. He also had behavioural issues, including mood disorder and aggressiveness (Table 1, Supplemental Material).

**Table 1:** Clinical features of the two families described in this study and previously reported individuals carrying *FGF14* deletions.

Study	Indiv	*FGF14* status	Affected status	Age at last examination (y)	Oculomotor anomalies		Neurological features					Neuroimaging	Development and psychiatric features
					Nystagmus	Others	Tremor	Ataxia	Balance	Other motor difficulties	Other	(MRI and/or CT)	
Current study - Family 1	I.1	DUP	A	31	LN	Asymmetric horizontal SmP	Yes	Yes (mild)	Poor	Fine motor difficulties		Normal	Mood disorder
	II.3	DUP	A	8	UN, horizontal GPN and RN	No horizontal and upward SmP, no vertical saccades, horizontal OKR asymmetry	Yes	Yes (mild)	Frequent falls	Fine/gross difficulties, dysmetria, DDK		Normal	Motor and speech delay, mood disorder, aggressiveness
Current study - Family 2	I.1	N/A	NE	Deceased	NE								
	I.2	WT	U	65	None								
	II.4	N/A	A	47	UN, GPN	SP					Seizures		
	II.6	DEL	A	46	UN, GPN	SP			Poor				
	II.7	N/A	U	43	None								
	II.8	DEL	A	40	UN, GPN				Poor				
	II.10	N/A	UA	37	None				Dizzy spells				
	II.11	WT	NE	36	NE								
	II.13	N/A	NE	35	NE								
	II.14	N/A	A	33	UN, GPN, RN	SP							
	II.15	N/A	U	31	None								
	II.16	DEL	A	54	UN, GPN	SP		Yes	Dizzy spells	Dysarthria (mild)			
	II.18	DEL	A	28	UN, GPN								
	III.21	N/A	A	26	GPN						Seizures		
	III.22	N/A	U	24	None								
	III.24	DEL	A	23	UN, GPN								
	III.25	N/A	U	12	None				Poor**				
	III.26	N/A	U	12	None				Poor**				
	III.27	WT	U	15	None								
	III.28	DEL	A	14	UN, DN, GPN, RN							Normal	
	III.29	DEL	A	32	UN, DN, GPN	SP		Yes	Dizzy spells			Normal	Borderline personality disorder, depression
	III.31	N/A	A	21	GPN, unsteady upgaze								
	III.32	N/A	NE	10	NE								
	III.33	WT	U	5									
	III.34	WT	U	25	None					Dyspraxia			Normal
	III.35	DEL	A	4	UN, GPN				Poor				
	III.36	DEL	A	1.5	GPN								
	III.46	WT	U*	NE	NE								
	III.63	DEL	A	17	Horizontal GPN	SP, dysmetric saccades			Normal				
	III.64	DEL	A	15	Horizontal GPN	SP, dysmetric saccades			Normal				
	IV.37	WT	U	7	None								
	IV.38	WT	U	3	None								
	IV.39	DEL	A	3	GPN	SP							
	IV.40	N/A	U	5	None								
	IV.65	WT	U*	NE	NE								
	IV.66	WT	U*	NE	NE								
	IV.81	N/A	A*	NE	NE								
Tucker et al. 2013	Proband	DEL	A	4.5	NR		Yes	Yes (mild)				Normal	IQ below average, speech delay, SE
Coebergh et al. 2014	Grand-mother	DEL	A	66	NR		Yes	Yes (mild)				Normal	Normal IQ
	Mother	DEL	A	NR	Yes		NR			No tandem walking		Normal	Normal IQ
	Proband	DEL	A	2	Horizontal and vertical GPN	SP, intrusive square wave jerks	No	Yes	Poor	Dysarthria, dysmetria, DDK		Normal	Normal IQ
Planes et al. 2015	Proband	DEL	A	20	NR	Delayed and slow saccades	Yes	Yes	Poor	1y: hypotonia, lower limb brisk tendon reflexes; 18 y: no tandem walking, dysmetria	Microcephaly	Atrophy (Crb, slowly progressive), cerebellar WM lesions	Moderate ID, speech delay
Amado et al. 2017	Adopted twins	DEL	A	4	Yes		Yes	Yes		Incoordination, dysarthria, dysmetria, DDK		Normal	Low IQ, memory and executive function impairment
Paucar et al. 2020	I:1	N/A	A	83	Yes		Yes	Yes	Poor	Dysarthria, dysmetria		Atrophy (Cor), WM anomalies	NE
	II:2	DEL	A	65	All directions	Weak horizontal and absent vertical OKN		Yes	Falls	Dysarthria, dysmetria, MPMC		NE	SE
	II:5	DEL	A	63	Yes	Weak horizontal and vertical OKN		Yes	Poor	Dysarthria, dysmetria, MPMC		Atrophy (Cor, Crb, Cen)	Low cognitive profile, SE
	III:1	DEL	A	48	Yes		Yes	Yes	Poor	Dysarthria, dysmetria	Hyporeflexia, polyneuropathy	Atrophy (Cor, Cen,Ver, Crb)	ID, SE, emotionally unstable personality disorder, psychosis, depression
	III:2	DEL	A	39	GPN	Weak vertical and horizontal OKN	Yes	Yes		Congenital cervical dystonia, dysmetria, MPMC		Atrophy (Cor,Ver, Csp)	Low IQ, language delay, dyslexia, SE, ADHD
	IV:1	DEL	A	18	Vertical and GPN	Weak vertical OKN	Yes	Yes		Dyspraxia, dysmetria, MPMC	Febrile seizures	Normal	Low cognitive profile, dyscalculia, SE, ADHD, anger outbursts
Zech et al. 2021	Proband	DEL	A	10	NR	NR		Yes		Childhood-onset segmental dystonia, myoclonus			

*A* Affected, *ADHD* Attention Deficit Hyperactivity Disorder, *Cen* Central, *Cor* Cortical, *Crb* Cerebellum, *Csp* Cervical spine, *CT* Computerised Tomography scan, *DDK* Dysdiadochokinesis, *DEL FGF14* deletion, *DN* Downbeat Nystagmus, *DUP FGF14* duplication, *GPN* Gaze evoked Nystagmus, *ID* Intellectual Disability, *IQ* Intelligence Quotient, *LN* Leftbeat Nystagmus, *MPMC* Minipolymyoclonus, *MRI* Magnetic Resonance Imaging, *N/A* Not Available, *NE* Not Examined, *NR* Not Reported, *OKN* Optokinetic Nystagmus, *OKR* Optokinetic Reflex, *RN* Rebound Nystagmus, *SE* Special Education, *SmP* Smooth Pursuit, *SP* Saccadic Pursuit, *U* Unaffected, *UA* Unassigned (examined but inconclusive symptoms/signs), *UN* Upbeat Nystagmus, *Ver* Vermis, *WM* White Matter, *WT* Wild-Type (no *FGF14* variant), *y* years. *Affected status reported by the family (not examined); **The twins had severe hearing loss, needing hearing aids, present since the neonatal period when they had severe complications requiring intensive care treatment. Their mild balance problems have been linked to these early difficulties.

His father (I.1) had poor balance, fine motor difficulties, and mood disorder. He had a history of tremor since childhood, initially attributed to asthma medication. He displayed mild left beating nystagmus in primary position, and eye movement recordings showed subtly asymmetric horizontal smooth pursuits. This was only evident on eye tracking with normal smooth pursuit response when moving the eyes to the left, but mildly reduced gain (the ratio of eye velocity to target velocity) when moving the eyes to the right. Neurological examination showed similar findings to the proband, including mild ataxia and mild intention tremor. His cranial MRI was normal (Table 1). The proband’s two sisters and mother had no medical problems.

**Fig. 2:**
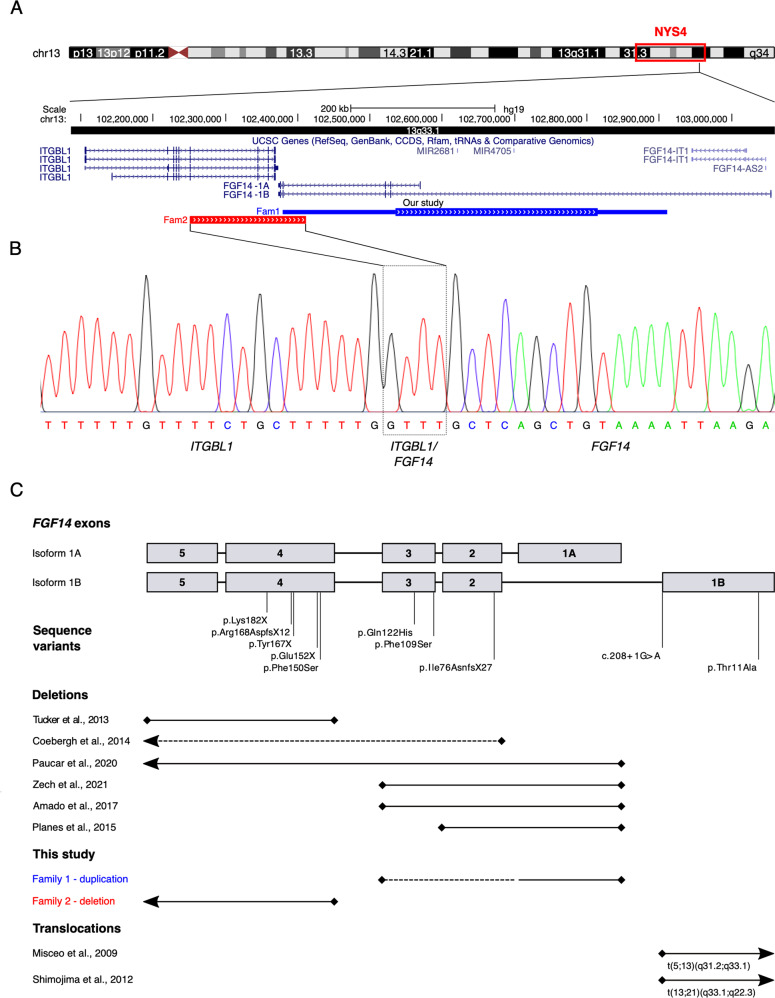
Characterisation of the two *FGF14* structural variants identified in families 1 and 2. **A** UCSC schematic (GRCh37, hg19) showing *ITGBL1* and *FGF14*. The blue bar indicates the region spanned by the duplication (family 1); the thicker region of the bar shows the minimum duplicated interval. The red bar indicates the region spanned by the deletion (family 2). **B** Sequence chromatogram showing the breakpoints of the deletion identified in family 2. The deleted region overlaps with 4/4 *ITGBL1* isoforms (including exons 8–11 in isoform 1, exons 7–10 in isoforms 2 and 3, and exons 7–11 in isoform 4) and the last two exons of *FGF14-1A/1B*. The 5′ boundary maps to an intronic region of *ITGBL1*, 114 bp from the nearest exon. The 3′ boundary maps to *FGF14* intron 3. The sequence GTTT is present at both ends of the CNV and therefore cannot be definitively ascribed to either side of the breakpoint. **C** Schematic of the two *FGF14* isoforms 1A and 1B indicating the location of structural and sequence variants identified in this study or previously reported in cases with SCA27/EA (see Supplemental Material for references). Sequence variants refer to *FGF14-1B* (NM_175929). Horizontal lines indicate the *FGF14* exons affected by structural variants. Arrows indicate variants extending to genes adjacent to *FGF14*, dashed bars indicate the exons affected by the maximum coordinates of the CNV. Note that the bars do not indicate the position of the breakpoints.

Array-CGH identified a partial *FGF14* duplication in both I.1 and II.3 between ~280 kb (chr13:102,535,482-102,815,349, hg19) and ~532 kb (chr13:102,379,344-102,911,282, hg19), which was absent from ClinVar (August 2022) and DECIPHER (April 15^th^ 2022 release). The two main isoforms of *FGF14*, *1A* (NM_004115) and *1B* (NM_175929), differ with respect to their first exon, with the minimum coordinates of the duplication encompassing at least exon 1 of isoform 1A (Fig. 2A). Read depth analysis of next-generation sequencing data from the father and seven normal controls suggests that exons 2–3 are also included in the duplication. If the duplication is in tandem, this would potentially lead to a frameshift in isoform 1B. Given that *FGF14* is a haploinsufficient gene, the CNV would therefore be classified as pathogenic [10]. Sequencing data confirmed the absence of pathogenic *FGF14* single nucleotide variants (SNVs) in the father.

### Family 2

The NYS4 pedigree [7, 8] now consists of 17 affected individuals with eye movement anomalies (Fig. 1B). These include nystagmus (gaze-evoked, upbeat and rebound), poor or absent smooth pursuit, and hyperactive vestibulo-ocular reflex. II.16 and III.29 also manifested ataxia, while II.6, II.8, and III.35 had balance problems. II.10 and III.34 reported dizzy spells and mild coordination problems, respectively, without nystagmus. Strabismus and seizures were variably present. Clinical features are summarised in Table 1.

WGS of III.63 and III.64 did not detect pathogenic SNVs in known nystagmus or ataxia genes. However, a 161 kb heterozygous deletion within the NYS4 interval was identified in both individuals (chr13:102,250,764-102,412,039, hg19), encompassing 2 exons of *FGF14* and 4–5 exons of *ITGBL1* (depending on isoform) (Fig. 2A, B). This CNV was also absent from ClinVar (August 2022) and DECIPHER (April 15th, 2022 release). Segregation analysis by PCR showed the deletion was present in 12/12 affected and 0/9 unaffected individuals (Table 1). The deletion was classified as pathogenic according to the ACMG guidelines [10].

## Discussion

We identified *FGF14* structural variants in two families with early onset nystagmus and variable neurological and behavioural features: a partial duplication of *FGF14* in a two-generation family and a heterozygous 161 kb deletion disrupting *FGF14* and *ITGBL1* in a previously described four-generation pedigree. These data finally elucidate the genetic variant underlying NYS4, a locus previously linked to the vestibulocerebellar condition described in the latter family.

*FGF14* encodes an intracellular fibroblast growth factor involved in multiple neuronal processes, including channel gating and neuronal excitability [11]. Individuals with pathogenic *FGF14* variants manifest EA or develop SCA27, a progressive cerebellar ataxia frequently presenting with nystagmus, tremor, dysarthria, limb ataxia, and variably associated with psychiatric symptoms and cognitive impairment. Eighteen pathogenic variants have been reported to date, including six heterozygous deletions [12–17], three of which overlap that of family 2 (Fig. 2C). While translocations and deletions are likely to cause functional haploinsufficiency, the effect of duplications is harder to predict. The variant in family 1 is the first report of a partial *FGF14* duplication and affects between one and three exons. Depending on the localisation and orientation of the duplicated fragment, this variant could alter the production, folding, localisation and/or function of the protein.

SCA27 is characterised by early onset and slow progression (ataxia onset: 23.7 ± 16.7 years), with only 13.8% of patients developing severe gait impairment [18]. In family 2, nystagmus was the most frequent and consistent feature, while balance problems were more variably present. Of note, four of five affected members exhibiting unsteadiness or ataxia were age ≥30 years at their last examination, whereas those not exhibiting ataxia/balance problems were mostly younger when examined [8]. Therefore, young age of assessment together with the variable presentation of ataxic features may account for the absence of gait impairment among family 2 carriers of the *FGF14* deletion.

Phenotypic intra- and inter-familial variability is a hallmark of *FGF14* variants [5, 18]. Family 2 expands this variability to include isolated nystagmus and milder clinical features. While III.63 had early onset nystagmus diagnosed by the age of three, her brother III.64 was initially reported as unaffected. Re-examination of III.64 on the basis of our genetic findings revealed a similar, but far more subtle, pattern of eye movement anomalies including horizontal gaze-evoked nystagmus, saccadic pursuit, and dysmetric saccades. Similarly, the affected status of II.10 was originally unassigned as she exhibited dizzy spells without nystagmus. While DNA was unavailable, the inheritance pattern of the deletion indicates that she is an obligate carrier, suggesting her phenotype represents an extremely mild form of SCA27. Therefore, family 2 supports an emerging model whereby mild phenotypes, including apparently isolated nystagmus, can result from variants in genes associated with ataxia [19].

Furthermore, this study highlights how detailed characterisation of oculomotor anomalies within a broader movement disorder can provide insights into the genetic basis of conditions such as SCA27. Early onset nystagmus with minimal or absent tremor and ataxia could be mistaken for other forms of nystagmus seen in infancy. In our families, the oculomotor pattern is mainly characterised by vertical nystagmus and horizontal gaze-evoked nystagmus with decelerating slow phases, which would be indicative of neurological nystagmus [20]. This supports some of the previous descriptions for *FGF14*-related conditions where details of eye movements are mentioned [12, 14]. However, since such detailed eye movement evaluation is rarely possible in routine clinical practice, particularly in children, we recommend the inclusion of *FGF14* on gene panels for childhood nystagmus.

In conclusion, our study identifies the genetic basis of NYS4, expands the spectrum of *FGF14* variants, refines the phenotypes of the associated oculomotor anomalies, and demonstrates the value of screening *FGF14* in children with apparently isolated early onset nystagmus.

## Supplementary information

Supplemental material

Dataset 1

## Data Availability

The two variants described in this study have been submitted to the ClinVar repository (SCV002570104, SCV002570105).
