# Supplementary material for: Analysis of Fibroblast Growth Factor 14 (FGF14) structural variants reveals the genetic basis of the early onset nystagmus locus NYS4 and variable ataxia
Source: Eur J Hum Genet. 2022 Oct 7;31(3):353–9. doi: 10.1038/s41431-022-01197-5 (PMC9995494; doi:10.1038/s41431-022-01197-5)
Supplement: Supplementary file 1 — Supplemental material [file 41431_2022_1197_MOESM1_ESM.docx]

**Supplemental Material**

**Phenotype description of family 1**

The proband is an eight-year-old boy, who presented to the eye clinic when he failed to meet a visual acuity of 0.2 logMAR at a school screening programme. He was born at full term, weighing 3400g after an uncomplicated pregnancy, the third child of non-consanguineous parents. He had developmental delay, walked after 2 years of age and had delayed speech, which had not been investigated. History taking at age 8 years revealed that at 2 years old his mother had noticed a fine tremor in both hands, which became more pronounced over time. At school he was noted to have difficulties with his motor skills. He struggled with fine motor skills showing poor handwriting, handling of cutlery and fastenings, and development of his gross motor skills, with difficulty with sporting activities and frequent falls.

At age 4 years the mother noticed that he had abnormal eye movements. Detailed orthoptic and ophthalmic examination at age 8 years revealed a pattern of oculomotor anomalies including vertical upbeat nystagmus in primary position, absent horizontal smooth pursuits and horizontal gaze evoked nystagmus with clear horizontal rebound nystagmus. A horizontal optokinetic reflex (OKR) asymmetry was seen between right and left stimuli with an absent response to a left stimulus, but present to a right stimulus. Vertical eye movements were impaired to a lesser degree than horizontal, with better smooth pursuit recordings; downward smooth pursuits were present with a reduced gain, but upward smooth pursuits were absent. OKR recordings showed similar findings whilst vertical saccades were absent. His irides were normally pigmented, and normal foveal architecture was seen on macular ocular coherence tomography (OCT). Electroretinogram (ERG) studies confirmed normal rod and cone function; monocular pattern and flash visually evoked potentials (VEPs) showed some possible crossed asymmetry at the optic chiasm.

Neurological examination showed bilateral intention tremor, mild dysmetria and dysdiadochokinesis. His gait was mildly affected with some difficulties with heel-to-toe walking. Cranial magnetic resonance imaging (MRI) was normal.

His father’s eye examination revealed a very subtle left beat nystagmus in primary position. His horizontal smooth pursuits showed a subtle asymmetry with normal smooth pursuit to the left but mildly reduced gain to the right, reflected in the OKR findings. Testing of saccades was clinically normal. The father’s neurological examination showed similar findings to those identified in the proband. Cranial MRI revealed no brain abnormality.

The proband’s two sisters and mother had no medical issues.

**Supplemental references**

The variants represented in Figure 2C have been reported in the following studies.

Amado, A., M. O. Blanco & A. Repáraz-Andrade (2017) Spinocerebellar Ataxia 27: Clinical Phenotype of Twin Sisters with FGF14 Deletion. *Neuropediatrics,* 48**,** 131.

Brusse, E., I. de Koning, A. Maat-Kievit, B. A. Oostra, P. Heutink & J. C. van Swieten (2006) Spinocerebellar ataxia associated with a mutation in the fibroblast growth factor 14 gene (SCA27): A new phenotype. *Mov Disord,* 21**,** 396-401.

Choi, K. D., J. S. Kim, H. J. Kim, I. Jung, S. H. Jeong, S. H. Lee, D. U. Kim, S. H. Kim, S. Y. Choi, J. H. Shin, D. S. Kim, K. P. Park, H. S. Kim & J. H. Choi (2017) Genetic Variants Associated with Episodic Ataxia in Korea. *Sci Rep,* 7**,** 13855.

Choquet, K., R. La Piana & B. Brais (2015) A novel frameshift mutation in FGF14 causes an autosomal dominant episodic ataxia. *Neurogenetics,* 16**,** 233-6.

Coebergh, J. A., D. E. Fransen van de Putte, I. N. Snoeck, C. Ruivenkamp, A. van Haeringen & L. M. Smit (2014) A new variable phenotype in spinocerebellar ataxia 27 (SCA 27) caused by a deletion in the FGF14 gene. *Eur J Paediatr Neurol,* 18**,** 413-5.

Coutelier, M., G. Coarelli, M. L. Monin, J. Konop, C. S. Davoine, C. Tesson, R. Valter, M. Anheim, A. Behin, G. Castelnovo, P. Charles, A. David, C. Ewenczyk, M. Fradin, C. Goizet, D. Hannequin, P. Labauge, F. Riant, P. Sarda, Y. Sznajer, F. Tison, U. Ullmann, L. Van Maldergem, F. Mochel, A. Brice, G. Stevanin, A. Durr & S. network (2017) A panel study on patients with dominant cerebellar ataxia highlights the frequency of channelopathies. *Brain,* 140**,** 1579-1594.

Dalski, A., J. Atici, F. R. Kreuz, Y. Hellenbroich, E. Schwinger & C. Zühlke (2005) Mutation analysis in the fibroblast growth factor 14 gene: frameshift mutation and polymorphisms in patients with inherited ataxias. *Eur J Hum Genet,* 13**,** 118-20.

Groth, C. L. & B. D. Berman (2018) Spinocerebellar Ataxia 27: A Review and Characterization of an Evolving Phenotype. *Tremor Other Hyperkinet Mov,* 8**,** 534.

Misceo, D., M. Fannemel, T. Barøy, R. Roberto, B. Tvedt, T. Jaeger, V. Bryn, P. Strømme & E. Frengen (2009) SCA27 caused by a chromosome translocation: further delineation of the phenotype. *Neurogenetics,* 10**,** 371-4.

Miura, S., K. Kosaka, R. Fujioka, Y. Uchiyama, T. Shimojo, T. Morikawa, A. Irie, T. Taniwaki & H. Shibata (2019) Spinocerebellar ataxia 27 with a novel nonsense variant (Lys177X) in FGF14. *Eur J Med Genet,* 62**,** 172-176.

Paucar, M., J. Lundin, T. Alshammari, Å. Bergendal, M. Lindefeldt, M. Alshammari, G. Solders, J. Di Re, I. Savitcheva, T. Granberg, F. Laezza, E. Iwarsson & P. Svenningsson (2020) Broader phenotypic traits and widespread brain hypometabolism in spinocerebellar ataxia 27. *J Intern Med,* 288**,** 103-115.

Piarroux, J., F. Riant, V. Humbertclaude, G. Remerand, J. Hadjadj, F. Rejou, C. Coubes, L. Pinson, P. Meyer & A. Roubertie (2020) FGF14-related episodic ataxia: delineating the phenotype of Episodic Ataxia type 9. *Ann Clin Transl Neurol,* 7**,** 565-572.

Planes, M., C. Rooryck, M. L. Vuillaume, L. Besnard, J. Bouron, D. Lacombe, B. Arveiler & C. Goizet (2015) SCA27 is a cause of early-onset ataxia and developmental delay. *Eur J Paediatr Neurol,* 19**,** 271-3.

Schesny, M., F. Joncourt & A. A. Tarnutzer (2019) Acetazolamide-Responsive Episodic Ataxia Linked to Novel Splice Site Variant in FGF14 Gene. *Cerebellum,* 18**,** 649-653.

Shimojima, K., A. Okumura, J. Natsume, K. Aiba, H. Kurahashi, T. Kubota, K. Yokochi & T. Yamamoto (2012) Spinocerebellar ataxias type 27 derived from a disruption of the fibroblast growth factor 14 gene with mimicking phenotype of paroxysmal non-kinesigenic dyskinesia. *Brain Dev,* 34**,** 230-3.

Tucker, M. E., F. M. Kalb & L. F. Escobar (2013) Infant Spinocerebellar Ataxia Type 27: Early Presentation Due To a 13q33.1 Microdeletion Involving the FGF14 Gene. *J Genet Syndr Gene Ther,* 4.

van Swieten, J. C., E. Brusse, B. M. de Graaf, E. Krieger, R. van de Graaf, I. de Koning, A. Maat-Kievit, P. Leegwater, D. Dooijes, B. A. Oostra & P. Heutink (2003) A mutation in the fibroblast growth factor 14 gene is associated with autosomal dominant cerebellar ataxia [corrected]. *Am J Hum Genet,* 72**,** 191-9.

Zech, M., S. Boesch, M. Škorvánek, J. Necpál, J. Švantnerová, M. Wagner, Y. Dincer, A. Sadr-Nabavi, T. Serranová, I. Rektorová, P. Havránková, S. Ganai, A. Mosejová, I. Příhodová, J. Šarláková, K. Kulcsarová, O. Ulmanová, K. Bechyně, M. Ostrozovičová, V. Haň, J. R. Ventosa, M. Shariati, A. Shoeibi, S. Weber, B. Mollenhauer, C. Trenkwalder, R. Berutti, T. M. Strom, A. Ceballos-Baumann, V. Mall, B. Haslinger, R. Jech & J. Winkelmann (2021) Clinically relevant copy-number variants in exome sequencing data of patients with dystonia. *Parkinsonism Relat Disord,* 84**,** 129-134.
